# Inflammatory and Oxidative Stress Markers—Mirror Tools in Rheumatoid Arthritis

**DOI:** 10.3390/biomedicines8050125

**Published:** 2020-05-15

**Authors:** Radu Răzvan Mititelu, Rodica Pădureanu, Manuela Băcănoiu, Vlad Pădureanu, Anca Oana Docea, Daniela Calina, Andreea Lili Barbulescu, Ana Maria Buga

**Affiliations:** 1 Department of Biochemistry, University of Medicine and Pharmacy of Craiova, 200349 Craiova, Romania; mradurazvan@gmail.com (R.R.M.); padureanurodica12@gmail.com (R.P.); 2 Department of Physical Therapy and Sports Medicine, University of Craiova, 200207 Craiova, Romania; manuelabacanoiu07@gmail.com; 3 Department of Internal Medicine, University of Medicine and Pharmacy of Craiova, 200349 Craiova, Romania; vlad.padureanu@umfcv.ro; 4 Department of Toxicology, University of Medicine and Pharmacy of Craiova, 200349 Craiova, Romania; daoana00@gmail.com; 5 Department of Clinical Pharmacy, University of Medicine and Pharmacy of Craiova, 200349 Craiova, Romania; calinadaniela@gmail.com; 6 Department of Pharmacology, University of Medicine and Pharmacy of Craiova, 200349 Craiova, Romania

**Keywords:** autoimmune diseases, rheumatoid arthritis, inflammation, oxidative stress, biomarkers

## Abstract

Rheumatoid arthritis (RA) is a chronic progressive autoimmune disease, associated with significant morbidity, mainly due to progressive damage and consequent disability. Oxidative stress is an important part of RA pathophysiology, as in autoimmune disease the interaction between immune response and endogenous/exogenous antigens subsequently induce the production of reactive oxygen species. The oxidative stress process seems to be positively strongly correlated with inflammation and accelerated joint destruction. We were asking ourselves if the oxidative stress biomarkers are the mirror tools of disease activity, outcome, and inflammation level in a group of RA patients under standard or biological therapy compared to healthy age-matched controls. In order to do this, the oxidative stress damage biomarkers (lipids peroxide and protein carbonyl level), antioxidant defense capacity, and pro-inflammatory status of plasma were quantified. In this study, we took into account the complete picture of RA diseases and assessed, for the first time, the inflammatory level in correlation with the oxidative stress level and antioxidant capacity of RA patients. Our results revealed that protein oxidation through carbonylation is significantly increased in RA groups compared to controls, and both protein carbonyl Pcarb and thiobarbituric acid reactive substance (TBARS) are reliable markers of ROS damage. Therefore, it is unanimous that neutrophil/lymphocyte ratio (NLR), monocyte/lymphocyte ratio (MLR), platelet/lymphocyte ratio (PltLR) correlated with Pcarb, and TBARS can provide a view of the complex phenomenon represented by proteins/lipids damage, key contributors to disease outcome, and an increased awareness should be attributed to these biomarkers.

## 1. Introduction

The pattern of autoimmune diseases implies an overactivation of the immune system, by different factors (e.g., genetic factors and/or environmental factors) and a switch mechanism between physiological and pathological status [1,2,3]. Rheumatoid arthritis (RA) is a chronic progressive autoimmune disease, associated with significant morbidity and an altered quality of life, mainly due to progressive damage and consequent disability. Despite multiple researches focused on oxidative stress biomarkers, only a few human studies were performed in order to assess the accuracy of these biomarkers in the clinical management of RA that should be easy to perform [4,5]. Several studies focused on genetic factors that can modulate the immune system, such as HLA, TNF or MTHFR gene polymorphism or, more recently identified, noncodant RNA molecules, that can be triggered by environmental chemical exposure [6,7]. There are also data describing the genetic polymorphisms that increase oxidative stress, which act as an intracellular signaling element and promote disease progression [5,8,9,10], by altering DNA, proteins, lipids, and stimulate the pathological process. Disease severity can be predicted by analyzing the histological aspects of synovial and extra-articular manifestations, including rheumatoid nodules, associated with rapid disease progression [11,12].

RA progression advances with the aging process, mainly due to systemic inflammation, and reactive oxygen species (ROS) overproduction can potentiate cells destruction along with a decreased antioxidant defense [13,14,15,16,17]. Oxidative stress is a hallmark of many diseases. The oxidative stress process seems to be strongly positively correlated with inflammation and accelerated joint destruction in RA patients [18,19,20]. Here, unlike the research studies, the clinical decision is not multimodal and does not address directly to a complex interaction between oxidative stress damage and pro-inflammatory status. A possible explanation can be the lack of accurate and easy to perform test and/or contradictory reports from little clinical studies. Many studies were focused on oxidative stress biomarkers in the animal model, but only a few clinical trials were performed in order to assess the accuracy of these biomarkers in the clinical management of RA patients [18,21,22,23,24,25]. Despite the fact that joint destruction consequences of RA diseases are a heterogenic disease with multiple patterns that can affect also, extra-articular tissues with a subtle response to disease modifying drugs (DMARDs) and evolution. The immune system is activated in RA and the peripheral blood monocyte, lymphocyte, neutrophil, and platelet cells are some key players [3,26,27]. However, the interrelation of the autoimmune process with oxidative stress in RA is still not fully understood. Therefore, we believe that it is crucial to understand the complex interrelation between oxidative stress and inflammatory biomarkers, in order to better predict the disease outcome and to prevent future articular and extra-articular damage associated with the ageing process.

There are not many data regarding the NLR, PLR, and MLR diagnostic value in autoimmune diseases and no studies were reported on the correlation between these biomarkers and oxidative stress level, therefore, we were focused on assessing oxidative stress biomarkers in a group of RA patients and their possible correlations with the disease activity, outcome, and inflammation level evaluated by some new potential markers (neutrophil/lymphocyte (NLR) ratio, monocyte/lymphocyte (MLR) ratio, and platelet/lymphocyte ratio (PltLR) [18,21,22,23,24,25]. Our objectives were to establish the oxidative alteration of proteins by carbonylation, alteration of lipids level by the thiobarbituric acid reactive substance (TBARS) as the end product of lipids peroxidation, to correlate the oxidative stress damage with a pro-inflammatory status using NLR, MLR, and PltLR as a new potential “in mirror tools”, and to establish if the total antioxidant capacity is adapted to the pro-oxidant and pro-inflammatory status of RA patients under standard or biological therapy.

## 2. Experimental Section

### 2.1. Ethical Issue

In this study, we included 15 patients with an active RA and 10 matched control subjects. The diagnosis of RA was established using the 2010 ACR/EULAR classification criteria for RA [28]. All the patients were nonsmokers, aged between 18 and 65 years and did not receive any supplements. 

All the patients that had other associated relevant systemic diseases that could affect the course of the study or restrict the patient ability to complete the study were excluded from the analysis. Healthy adult volunteers were recruited from the staff of hospital and university departments. Eligibility was confirmed through screening assessments such as medical history (no history of autoimmune diseases or clinically significant comorbidities), physical examination (vital signs, ECG), and basic laboratory tests (CBC test, inflammatory biomarkers, and standard biochemical tests). We subdivided the RA groups in two subgroups, treated with standard and combined therapy. RA group 1 received standard therapy with leflunomide (LEF) or methotrexate (MTX) and RA group 2 received combined therapy with a synthetic disease modifying antirheumatic drug DMARDs (MTX or LEF) and biologic DMARDs (adalimumab or etanercept) for 12 weeks. This study was approved by the University of Medicine and Pharmacy of Craiova ethics committee (No. 32174/2019; 96/2019). All selected participants gave their written informed consent to participate in the study. Exclusion criteria were comorbidities that are associated with increased inflammatory status (e.g., diabetes mellitus, other autoimmune diseases, malignancy), smokers, or pregnancy.

### 2.2. Sample Collection

The venous blood fasting (no food or drinks were allowed at least 12 h before) samples were collected by an experimented phlebotomist. Blood samples were collected on commercially available EDTA (ethylene-diamine-tetraacetic acid, Becton Dickinson, U.S.) tubes and was used to perform complete blood counting (CBC). For plasma collection, blood samples were collected on sodium citrate commercially available tubes. Blood cells were separated from plasma by centrifugation at 2000× *g* for 10 min in a refrigerated centrifuge (5417R Eppendorf, Eppendorf AG, Germany). Blood cells were removed from the plasma immediately following centrifugation. Plasma aliquots were properly stored (at −80 °C, with avoiding repeated freezing/refreezing cycle) for further analysis of oxidative stress markers, these are in accord with other studies that showed the sample stability for oxidative stress markers in biological fluids (for 1 year at −80 °C or two days at 4 °C) [29,30]. For inflammatory markers, venous blood samples were collected on a vacutainer from Becton Dickinson (BD) and the blood was allowed to clot for 20 min at room temperature (RT). The clot was removed by centrifugation at 2000× *g* for 10 min in a refrigerated centrifuge (5417R Eppendorf, Eppendorf AG, Germany) and the serum sample was used for an analysis of inflammatory markers. In order to avoid analytical errors only the optimum sample was analyzed (fifteen samples in the experimental group and ten in the control). Haemolyzed samples (four samples from the control and one sample from the PR group) were excluded from the analysis.

### 2.3. Oxidative Stress Markers Analysis Steps

Protein carbonyl analysis (Pcarb) was performed by a spectrophotometric method, based on the carbonyl reagent reaction, 2,4-dinitrophenylhydrazine (DNPH or Brady’s reagent, Sigma Aldrich, Germany). This method is one of the easiest tools to perform and a successfully used method in order to analyze the Pcarb content, similar to separation by chromatographic methods as gas chromatography (GC) or high-performance liquid chromatography (HPLC) [31,32,33,34]. The laboratory procedure for protein carbonyl analysis followed some steps. First, the plasma proteins were precipitated with an equal volume of trichloroacetic acid 20% (TCAA, Sigma Aldrich, Germany) diluted in distillate water. In this stage, the mixtures were stored on ice for 15 min. After the incubation step, the samples were centrifuged for 5 min at 15,000× *g* in a centrifuge with a cooling system (Eppendorf 5417R, Eppendorf AG, Germany) and 0.01 M of the DNPH reagent was added on the precipitate. The protein precipitate in the DNPH reagent was stored in a dark chamber at RT for 1 h. After this stage, the upper phase was removed and three washing steps with a solution of ethanol-ethyacetate (Sigma Aldrich, Germany) were performed before solving the protein precipitate in a 5 M urea pH = 2.3 (Sigma Aldrich, Germany) at 37 °C for 15 min. The samples were centrifuged and a clear supernatant was further performed by reading the absorbance at 375 nm. Analytical performance of the Pcarb analysis was between 0.1–5 nmol/mg of protein (linear range). The Pcarb level was extracted from all protein levels (established using the Bradford reagent method) in a rapport with the molar extinction coefficient of DNPH (22 mM^−1^·cm^−1^) [31,32,33,34].

### 2.4. Lipid Peroxidation Analysis

The lipid peroxidation analysis was established by quantifying TBARS using a spectrophotometric method. The TBARS assay is the most common used method to measure the level of malondialdehyde (MDA), the major lipid oxidation product in biological fluids, cell extract, and tissue sample, and correlated with other oxidative stress markers can offer a valuable window on the complex phenomenon of lipid peroxidation [29,32,33,34]. The TBARS testing principle is based on the properties of thiobarbituric acid to react with the lipids hydroperoxide (Sigma Aldrich, Germany) and generate malondialdehyde that are generated from the lipid peroxidation reaction. In order to perform this analysis several steps are required as follows: The plasma sample was added on the TCAA/Tris-HCL (Sigma Aldrich, Germany) mixture with pH = 7 for 10 min at room temperature (RT). After the incubation time, the samples were heated at 95 °C for 45 min with a reagent containing a 0.05 M thiobarbituric acid (Sigma Aldrich, Germany) and 2 M sodium sulphate (Sigma Aldrich, Germany). After cooling, the mixture can be centrifuged at 15,000× *g* for 3 min in a centrifuge with a cooling system (Eppendorf 5417R, Eppendorf AG, Germany). Malondialdehyde reacts with the thiobarbituric acid and forms a pink color product with a specific absorbance wavelength between 532 and 535 nm. Analytical performance of the TBARS analysis was between 0.1–10 µmol/L (linear range). The TBARS concentration was extracted based on the molar extinction coefficient of MDA (1.55 × 105 M^−1^ cm^−1^). The TBARS level was expressed in µmol/L [31,34,35,36].

### 2.5. Total Antioxidant Capacity (TAC) Analysis

Total antioxidant capacity (TAC) analysis is the capacity of the human plasma to maintain the levels of ROS that were measured using spectrophotometric methods with a DPPH reagent (Sigma Aldrich, Germany). The TAC assay is a common, rapid, and simple method to measure the antioxidant capacity of biological fluids. Several methods are available to measure TAC in plasma but the basic approach is similar for all of them [31,34,37]. The method is based on the ability of the plasma sample to oxidize the DPPH, a high purple color compound that became discolored. The laboratory procedure for TAC follows some steps. The dilutions of the plasma sample in a 1 × phosphate buffer saline (PBS, Sigma Aldrich, Germany) are added in a 0.01 M DPPH reagent (*v*/*v*) and stored for 30 min in a dark chamber. After the incubation time, the samples were centrifuged at 20.000× *g* for 3 min in a centrifuge with a cooling system (Eppendorf 5417R, Eppendorf AG, Germany). The absorption of samples was read at 517 nm using a Hitachi UV–VIS spectrophotometer (Hitachi High-Tech Science Corporation, Tokyo, Japan). Analytical performance of the TAC analysis was between 0–1100 µmol ascorbic acid (linear range). TAC was established using the molar extinction coefficient of DPPH (11,500 M^−1^·cm^−1^) [31,34,37]. 

### 2.6. Complete Blood Counting (CBC)

In order to perform complete blood counting (CBC) we used a five-part differential cell counter that used both flow-cytometry and Coulter’s principle (Ruby Cell-Dyne, Abbott, USA) to differentiate all five types of peripheral blood cells (neutrophils, monocytes, lymphocytes, basophils, and platelets). We extracted NLR, MLR, and PltLR using these hematological measurements [31,38].

Moreover, the erythrocyte sedimentation ratio (ESR) was assessed according to the Westergren method (ESR tubes, Becton Dickinson, USA). The C-reactive protein (CRP) markers analysis using an automated assay analyzer was based on the chemiluminescence immunoassay method (Cobas e411, Roche Diagnostics GmbH, Mannheim, Germany). We recorded the following general patient information: Age, sex, and disease progression time. Furthermore, we recorded the specific disease severity assessment using the DAS 28 score, the patient global assessment of disease activity using a visual analog scale (patient global VAS), and the original health assessment questionnaire (HAQ) used in rheumatology was used to establish the HAQ score.

### 2.7. Statistical Analysis

Data were analyzed using the GraphPad Prism 5.0 software (GraphPad Software, San Diego, CA, USA). The results are calculated as mean ± standard deviation (SEM). The significant differences between studied groups for normally distributed data were analyzed by the unpaired Mann–Whitney *t*-test. The nonparametric Pearson’s test was calculated to test the correlation between biological and clinical variables (DAS28), with a receiver operating characteristic (ROC) curve for sensitivity, and specificity with an area under the ROC curve (AUC) for accuracy of the tests. *p*-values less than 0.05 (*p* ≤ 0.05) were established as significant changes.

## 3. Results

We included 15 RA patients (about 38% of the RA patients practiced during the period of study), according to the 2010 ACR/EULAR classification criteria [28] and 10 aged-matched healthy subjects, without history of an autoimmune or inflammatory disease. The healthy volunteer subjects were selected based on screening assessments (medical history, physical examination, basic laboratory tests, vital signs, ECG, and check of inclusion and exclusion criteria). Demographic and clinical characteristics are presented in Table 1.

Biological and clinical characteristics of RA subgroups are presented in Table 2.

### 3.1. The Immune Status Biomarkers

#### 3.1.1. Neutrophil/Lymphocyte Ratio (NLR)

**Figure 1:**
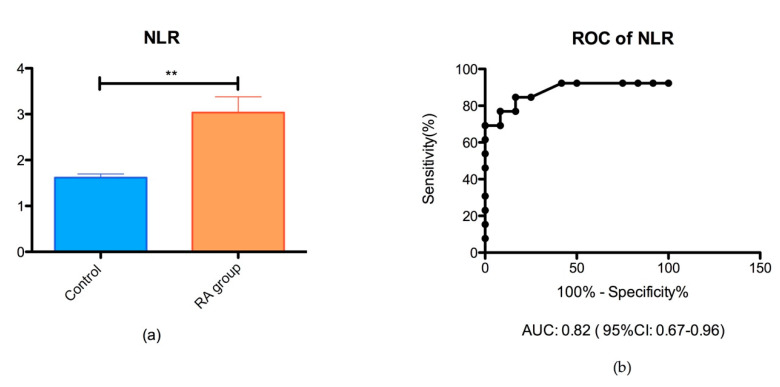
(**a**) Bar plot (mean with SEM) of the neutrophil/lymphocyte (NLR) ratio in the rheumatoid arthritis (RA) group vs. control group (** *p* = 0.001); (**b**) receiver operating characteristic (ROC) analysis of NLR in the RA group vs. control group (AUC = 0.82).

The NLR ratio was significantly different in the groups of RA patients (3.03 ± 0.35) than controls (1.62 ± 0.28) (** *p* < 0.05), as shown in Figure 1. 

#### 3.1.2. Monocyte/Lymphocyte Ratio (MLR)

**Figure 2:**
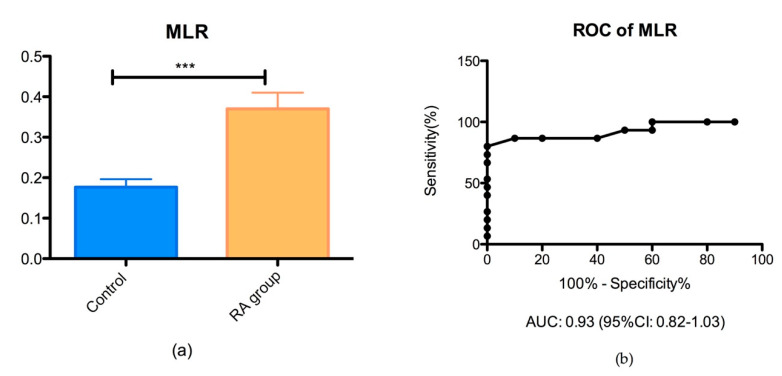
(**a**) Bar plot (mean with SEM) of the monocyte/lymphocyte (MLR) ratio in the RA group vs. control group (*** *p* = 0.0004); (**b**) ROC analysis of MLR in the RA group vs. control group (AUC = 0.93).

The MLR ratio was significantly different in the groups of RA patients (0.37 ± 0.16) than controls (0.17 ± 0.06) (*** *p* < 0.05), as shown in Figure 2.

#### 3.1.3. Platelet/Lymphocyte Ratio (PltLR)

**Figure 3:**
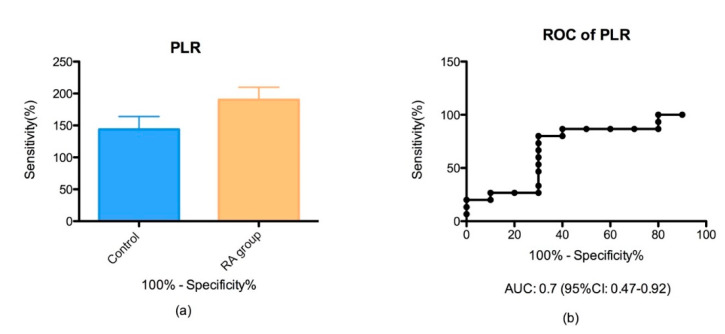
(**a**) Bar plot (mean with SEM) of the platelet/lymphocyte ratio (PltLR) in the RA group vs. control group (*p* = 0.001); (**b**) ROC analysis of PltLR in the RA group vs. control group (AUC = 0.70).

PltLR showed an AUC of 0.7% and 95% CI between 0.47–0.92 and was slightly increased in the RA group (143.8 ± 20.22) compared to the control group (190.2 ± 19.63) but we did not find a major difference between groups (*p* > 0.05) (Figure 3).

### 3.2. Oxidative Stress Products in Plasma

**Figure 4:**
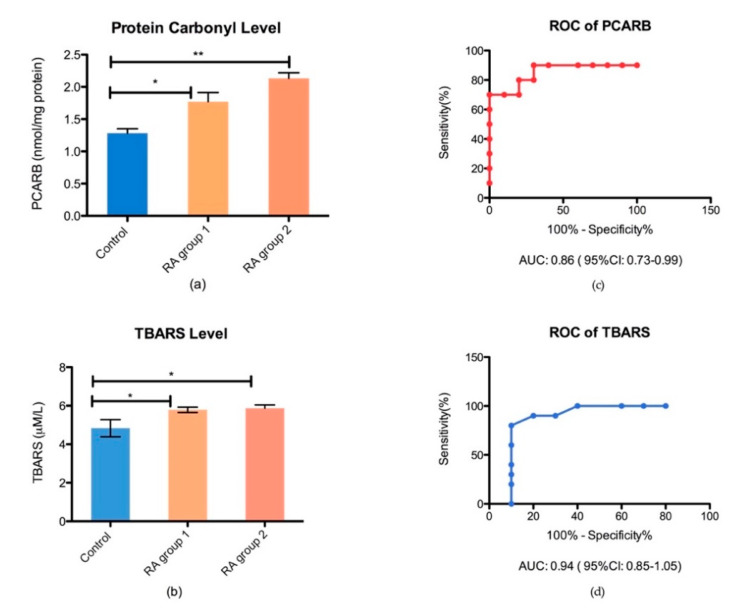
Bar plot (mean ± SEM) and ROC analysis of oxidative stress biomarkers in plasma, (**a**) protein damage marker in plasma (Pcarb) in healthy controls vs. RA group 1 (* *p* < 0.05) and RA group 2 (** *p* < 0.05); (**b**) lipid peroxidation marker in plasma (TBARS) in healthy controls vs. RA group 1 (* *p* < 0.05) and RA group 2 (* *p* < 0.05); (**c**) ROC analysis of TBARS performance in plasma; (**d**) the ROC analysis of Pcarb performance in plasma.

The end-product of ROS damage, such as protein carbonyl levels (Pcarb) for protein damage and thiobarbituric acid reactive substance (TBARS), for lipid peroxidation damage in the plasma were assessed in controls and RA subgroups. We found an increased Pcarb level for RA patients compared to controls (*p* < 0.05). However, there was not any significant difference between the RA subgroups, treated with synthetic vs. combined therapy (Figure 4). The receiver operator characteristic (ROC) analysis with an area under the curve (AUC) and 95% confidence interval (CI) was performed in order to establish the plasmatic oxidative stress biomarkers performance (Figure 4).

### 3.3. Antioxidant Defense

**Figure 5:**
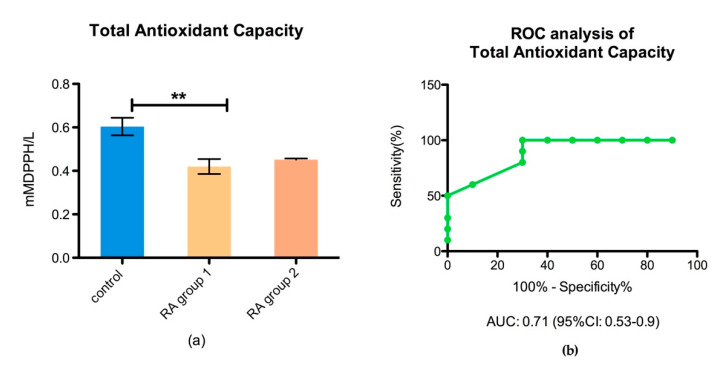
Bar plot (mean ± SEM) and ROC analysis of the total antioxidant capacity in plasma, (**a**) total antioxidant capacity (TAC) in healthy controls vs. RA group 1 (*p* < 0.05) and RA group 2 (** *p* < 0.05); (**b**) ROC analysis of TAC performance in plasma.

The total antioxidant capacity (TAC) of the body, for controls and RA patient subgroups, as well as the performance of the total antioxidant capacity test, was analyzed by ROC, AUC, and 95% of CI (Figure 5).

### 3.4. Correlation of Oxidative Stress with Inflammation

**Figure 6:**
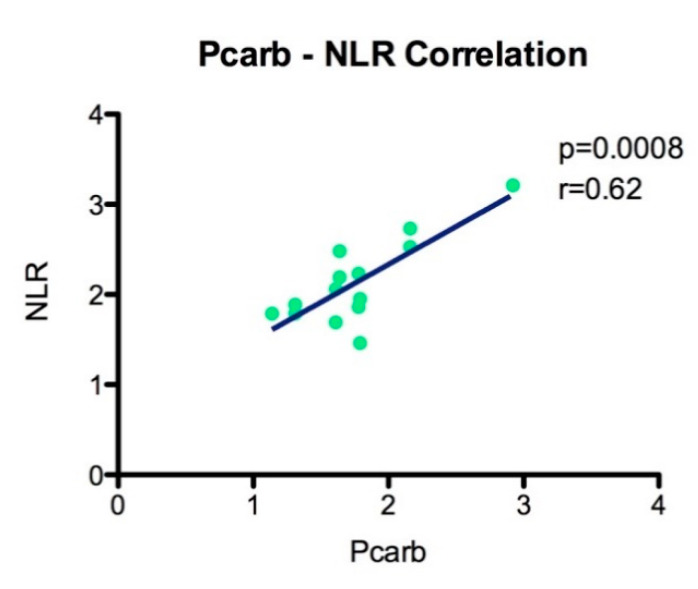
Association analysis of the Pcarb level with NLR (*p* = 0.0008, r = 0.62).

Using Pearson’s correlation analysis and linear regression, we showed that the protein carbonyl level (Pcarb) is statistically significant and positively correlated with NLR (*p* = 0.0008, r = 0.62) (Figure 6).

## 4. Discussion

In patients with RA, an early therapeutic intervention can prevent future damage and complications, improving the quality of life, but a percentage of patients undergoing immunosuppressive therapy, synthetic, biologic or combination, can experience at least a flare during disease evolution [39,40]. Our objectives were focused on new potential biomarkers associated with an increased pro-inflammatory status and oxidative stress that are easy to perform, reproducible, and can improve disease management. Recent scientific reports described NLR, MLR, and PltLR as new biomarkers associated with inflammatory status and disease activity, with an increased accuracy and availability [31,41,42]. The platelet/lymphocyte ratio (PltLR) and neutrophil/lymphocyte ratio are mirror tools for the immune system status of the body in autoimmune diseases that are characterized by an overactivation of the inflammatory cascade. A major need in RA management is to discover noninvasive tests that can be used to monitor the immune status of the body in RA patients, we asked if these biomarkers are reliable for this purpose. However, NLR and PltLR were described to be significantly altered in many other pathological conditions such as cancers, osteoarthritis, ischemic stroke, or myocardial infarction [43,44,45,46,47]. Only a few studies have reported the NLR, PltLR, and MLR as measurement tools of inflammation in rheumatoid arthritis and reported that these markers are correlated with DAS28, ESR, and CRP [48,49,50,51,52]. However, no other previous studies showed the complete picture of RA pattern taking into account the inflammatory level in correlation with the oxidative stress level and antioxidant capacity of RA patients.

Our data show that NLR is significantly higher in the RA group compared to controls (*p* = 0.001) such as the previous reports, proving a consensus of the NLR pattern in RA diseases [49,53]. An interesting result is that we found NLR to be a feasible marker for pro-inflammatory status, when the cutoff value is set at 2.6 with an AUC of 0.820 (95% CI of 0.67–0.96) according to the ROC analysis. However, we did not find a significant difference between the RA subgroups, meaning that NLR is not strongly influenced by a standard therapy with leflunomide (LEF) or methotrexate (MTX) or by a combined therapy with a synthetic disease modifying antirheumatic drug DMARDs (MTX or LEF) and biologic DMARDs (adalimumab or etanercept).

Moreover, it is well known that monocytes can be triggered by the inflammasome signaling pathway and are actively involved in RA disease activity. Previous studies showed that the ROS act as a trigger that switch on the NLRP3 inflammasome and contribute to disease progression in a vicious cycle [54]. In accord with previous studies, we found that MLR increased in RA patients compared to the healthy-matched group [29]. Our study revealed that MLR has an increased accuracy compared to NLR, to an AUC of 0.93, and 95% CI between 0.82 and 1.03, as a result of ROC analysis. These results prove that the MLR has a considerable value for RA management.

In our study, we did not find a significant difference of PltLR between groups, a possible explanation for this result can be the small number of patients included in this study. Moreover, we found that PltLR has a moderate accuracy compared to NLR and MLR, to an AUC of 0.7 and 95% CI between 0.47 and 0.92, that is in accord with the previous study [42].

In consent with previous studies, we found that NLR and PltLR are positively correlated with the disease activity score (DAS28) [35,36,37,38,39]. NLR was significantly correlated with DAS28 (*p* = 0.025; r = 0.44) with a 95% CI between 0.1 and 0.9. PltLR was significantly correlated with DAS28 (*p* = 0.01; r = 0.43) with a 95% CI between 0.19 and 0.88.

Oxidative stress is an important part of RA pathophysiology together with inflammation, as in autoimmune disease the interaction between immune response and endogenous/exogenous antigens subsequently induce the production of reactive oxygen species. An increased level of oxidative stress level is considered a hallmark of the normal aging process that can influence both the response to therapy and disease progression. However, the interrelation between inflammatory and oxidative stress biomarkers is not completely understood. Despite recent progress in the oxidative stress research area, a clear link between free radicals and diseases is far to be clarified. This can be caused by the complex interaction of the environmental factors, ageing progression that change a basal status of the body, or due to a complex molecular and cellular mechanisms of oxidative stress in different contexts such as inflammation. Hence, an important question is whether the evaluation of a basic oxidative stress level is mandatory and accurate for assessing therapeutic response during the aging period. Therefore, we determined oxidative stress markers of protein and lipid damage in a group of RA patients, undergoing synthetic and biologic DMARD therapy, compared to a healthy age-matched group. Our results revealed that protein oxidation through carbonylation (Pcarb level) is significantly increased in RA groups compared to controls (*p* < 0.05), with no significant difference between RA subgroups (a possible explanation being the relative low number of patients). Moreover, the lipid peroxidation (LPO) level, measured by TBARS, was statistically significantly higher in RA patients (*p* < 0.05). Both, Pcarb (AUC = 0.86 ± 0.1; 95% CI between 0.73 and 0.99) and TBARS are reliable markers of ROS damage, with an excellent accuracy for the TBARS assay (AUC = 0.96 ± 0.09; 95% CI between 0.85 and 1.05). Furthermore, we found that Pcarb and the lipid peroxidation marker are significantly correlated with the disease activity (DAS28). The Pcarb level was significantly correlated with DAS28 (*p* = 0.009; r = 0.44) with a 95% CI between 0.1 and 0.9. PltLR was significantly correlated with DAS28 (*p* = 0.01; r = 0.43) with a 95% CI between 0.19 and 0.89.

An important role in antioxidant defense is attributed to the redox balance, between oxidative and antioxidant compounds. However, the oxidative stress damage is a dynamic process that involves not only the changes in ROS production and increased oxidative damage, but also the ability of the body to counteract the ROS level by an antioxidant capacity in order to limit the oxidative damage. The individual variability in oxidative stress response to diseases should not be ignored. It is well known that we can face an increased ROS production for a short period of time as soon as we pose the efficient antioxidant capacity. We can have an opposite situation, when even at a low level of ROS production an increased oxidative stress damage can be recorded due to a deficient antioxidant capacity of the body. In this light, it is crucial to assess the oxidative stress level from these two perspective pro-oxidative and anti-oxidative levels, in order to achieve clinically relevant results. We showed that the antioxidant capacity of the body (TAC) was decreased in all RA groups compared to controls and did not face an increased level of oxidative stress. Moreover, our data demonstrated that, for RA patients, the TAC level was a biomarker with an increased accuracy (AUC of 0.71 and 95% CI between 0.53 and 0.9) in assessing antioxidant capacity. However, we showed for the first time that the protein damage biomarker Pcarb was highly correlated with NLR and these findings can be a valuable tool in the management of RA diseases.

Our study has some limitations, first of all, represented by the relatively small number of subjects, but it is a starting point for our research and requires its extension, by performing a multicenter analysis. Moreover, we did not address the molecular mechanism that underlies the NLR, MLR, and PltLR changes that can lead to oxidative stress damage in a vicious cycle. However, there are other processes that are involved many signaling pathways such as apoptosis and cell death, cell division and proliferation, immune and inflammatory response, as well as metabolism and cellular homeostasis. These aspects need further investigations. Further data regarding correlations between inflammatory and oxidative stress biomarkers, that are easily detectable, will be clearer and will help us establish the key factors with a significant input on the management of RA patients, in order to enable the diagnosis, to obtain a valid target strategy that can improve the prognosis and quality of life for these patients.

## 5. Conclusions

Oxidative stress is an important contributor when establishing therapeutic options for RA patients, especially in an older population, with a long duration of the disease and risk of high of progression. An optimal management of these patients involves determining certain biomarkers, with a high accuracy and feasibility, which can reflect the complex interrelation between inflammatory status and oxidative stress level. It is unanimous that the inflammatory mirror NLR, MLR, and PltLR together with Pcarb and TBARS levels as an oxidative stress mirror can provide a view of the complex phenomenon represented by proteins/lipids damage, key contributors to disease outcome, and an increased awareness should be attributed to these biomarkers. In this study, we took into account, for the first time the inflammatory level in correlation with the oxidative stress level and antioxidant capacity of RA patients, as a complete picture of the status of RA diseases. Finally, we conclude that NLR, MLR, PltLR correlated with Pcarb, and TBARS can provide a view of the complex phenomenon represented by proteins/lipids damage, key contributors to the disease outcome, and an increased awareness should be attributed to these biomarkers.

## Figures and Tables

**Table 1:** Demographic and clinical characteristics.

Characteristics	RA Group	Control Group
Age mean	59.69 ± 8.53	56.4 ± 6.73
Sex ratio (F:M)	11:4	7:3
DAS28	2.95 ± 0.7	-
Patient global VAS (mm)	37.69 ± 13.63	-
ESR (mm/h)	30.6 (5–125) ^1^	8.4 (7–8)
CRP (mg/dl)	6.71 (1.4–66.9) ^1^	2.5 (0–2.5)
HAQ	0.48 (0.1–0.9)	-

^1^ These results were significantly higher than controls (*p* < 0.05) (DAS28: Disease Activity Score 28; VAS: Visual Analog Scale; ESR: Erythrocyte Sedimentation Rate; CRP: C-Reactive Protein; HAQ: Health Assessment Questionnaire).

**Table 2:** Biological and clinical characteristics in rheumatoid arthritis (RA) subgroups.

Characteristics (Mean ± SEM)	RA Subgroup 1	RA Subgroup 2
DAS28	3.09 ± 0.2	2.49 ± 0.7
Global VAS (mm)	41.0 ± 0.3 ^1^	26.67 ± 1.8
ESR (mm/h)	36.8 (5–125)	15.8 (6–27)
CRP (mg/dl)	6.75 (0.4–66.9)	6.7 (3–9)
HAQ	0.55 (0.1–1.1)	0.25 (0.1–0.3)
NLR	3.02 ± 0.42	3.47 ± 0.12
MLR	0.38 ± 0.2	0.34 ± 0,15
PltLR	206.94 ± 86.03	134.5 ± 33.2

^1^ These results were significantly higher than controls (*p* < 0.05) (NLR: Neutrophil/lymphocyte ratio; MLR: Monocyte/lymphocyte ratio; PltLR: Platelet/lymphocyte ratio).

## References

[1] Scrivo R., Perricone C., Altobelli A., Castellani C., Tinti L., Conti F., Valesini G. (2019). Dietary Habits Bursting into the Complex Pathogenesis of Autoimmune Diseases: The Emerging Role of Salt from Experimental and Clinical Studies. Nutrition.

[2] Fousert E., Toes R., Desai J. (2020). Neutrophil Extracellular Traps (NETs) Take the Central Stage in Driving Autoimmune Responses. Cells.

[3] De Rasmo D., Ferretta A., Russo S., Ruggieri M., Lasorella P., Paolicelli D., Trojano M., Signorile A. (2020). PBMC of Multiple Sclerosis Patients Show Deregulation of OPA1 Processing Associated with Increased ROS and PHB2 Protein Levels. Biomedicines.

[4] Mun S., Lee J., Park A., Kim H.-J., Lee Y.-J., Son H., Shin M., Lim M.-K., Kang H.-G. (2019). Proteomics Approach for the Discovery of Rheumatoid Arthritis Biomarkers Using Mass Spectrometry. Int. J. Mol. Sci..

[5] Long N.P., Park S., Anh N.H., Min J.E., Yoon S.J., Kim H.M., Nghi T.D., Lim D.K., Park J.H., Lim J. (2019). Efficacy of Integrating a Novel 16-Gene Biomarker Panel and Intelligence Classifiers for Differential Diagnosis of Rheumatoid Arthritis and Osteoarthritis. J. Clin. Med..

[6] Docea A.O., Gofita E., Goumenou M., Calina D., Rogoveanu O., Varut M., Olaru C., Kerasioti E., Fountoucidou P., Taitzoglou I. (2018). Six months exposure to a real life mixture of 13 chemicals’ below individual NOAELs induced non monotonic sex-dependent biochemical and redox status changes in rats. Food Chem. Toxicol..

[7] Wahba A., Ibrahim M., Mesbah N., Saleh S., Abo-Elmatty D., Mehanna E. (2020). Serum LINC00305 expression and its genetic variant rs2850711 are associated with clinical and laboratory features of rheumatoid arthritis. Br. J. Biomed. Sci..

[8] Tanhapour M., Shahmohamadnejad S., Vaisi-Raygani A., Kiani A., Shakiba Y., Rahimi Z., Bahrehmand F., Shakiba E., Vaisi-Raygani A.-A., Alibakhshi R. (2018). Association between activity and genotypes of paraoxonase1 L55M (rs854560) increases the disease activity of rheumatoid arthritis through oxidative stress. Mol. Boil. Rep..

[9] Shahmohamadnejad S., Vaisi-Raygani A., Shakiba Y., Kiani A., Rahimi Z., Bahrehmand F., Shakiba E., Pourmotabbed T., Shahmohamdnejad S. (2015). Association between butyrylcholinesterase activity and phenotypes, paraoxonase192 rs662 gene polymorphism and their enzymatic activity with severity of rheumatoid arthritis: Correlation with systemic inflammatory markers and oxidative stress, preliminary report. Clin. Biochem..

[10] Rogoveanu O.C., Calina D., Cucu M.G., Burada F., Docea A.O., Sosoi S., Stefan E., Ioana M., Burada E. (2018). Association of cytokine gene polymorphisms with osteoarthritis susceptibility. Exp. Ther. Med..

[11] Trăistaru M.R., Kamal D., Traşcă D.M., Foarfă M.C., Gruia C.L., Rogoveanu O.C. (2016). Rheumatoid nodules and quality of life in rheumatoid arthritis females—Complex assessment. Rom. J. Morphol. Embryol..

[12] Florentin-Ananu V., Chisalau B., Parvanescu C., Barbulescu A., Rogoveanu O., Firulescu S., Rosu A., Ciurea P. (2016). High Frequency Ultrasonography of the Hand in Rheumatoid Arthritis, Psoriatic Arthritis, Gout and Osteoarthritis Patients. Curr. Heal. Sci. J..

[13] Mateen S., Moin S., Khan A.Q., Zafar A., Fatima N. (2016). Increased Reactive Oxygen Species Formation and Oxidative Stress in Rheumatoid Arthritis. PLoS ONE.

[14] Phull A.R., Nasir B., Haq I.-U., Ashraf Z. (2018). Oxidative stress, consequences and ROS mediated cellular signaling in rheumatoid arthritis. Chem. Interact..

[15] Goronzy J.J., Shao L., Weyand C.M. (2010). Immune Aging and Rheumatoid Arthritis. Rheum. Dis. Clin. North Am..

[16] Lindstrom T.M., Robinson W.H. (2010). Rheumatoid arthritis: A role for immunosenescence?. J. Am. Geriatr. Soc..

[17] Chalan P., Berg A.V.D., Kroesen B.-J., Brouwer L., Boots A. (2015). Rheumatoid Arthritis, Immunosenescence and the Hallmarks of Aging. Curr. Aging Sci..

[18] Da Fonseca L.J.S., Nunes-Souza V., Goulart M.O.F., Rabelo L.A. (2019). Oxidative Stress in Rheumatoid Arthritis: What the Future Might Hold regarding Novel Biomarkers and Add-On Therapies. Oxidative Med. Cell. Longev..

[19] Smallwood M.J., Nissim A., Knight A.R., Whiteman M., Haigh R., Winyard P. (2018). Oxidative stress in autoimmune rheumatic diseases. Free Radic. Boil. Med..

[20] Sies H., Berndt C., Jones D.P. (2017). Oxidative Stress. Annu. Rev. Biochem..

[21] García-González A., Gaxiola-Robles R., Zenteno-Savin T. (2015). Oxidative stress in patients with rheumatoid arthritis. Rev. Invest. Clin..

[22] Quiñonez-Flores C.M., González-Chávez S.A., Nájera D.D.R., Pacheco-Tena C. (2016). Oxidative Stress Relevance in the Pathogenesis of the Rheumatoid Arthritis: A Systematic Review. Biomed. Res. Int..

[23] Vaghef-Mehrabany E., Rad A.H., Alipour B., Sharif S.-K., Vaghef-Mehrabany L., Alipour-Ajiry S. (2015). Effects of Probiotic Supplementation on Oxidative Stress Indices in Women with Rheumatoid Arthritis: A Randomized Double-Blind Clinical Trial. J. Am. Coll. Nutr..

[24] Batooei M., Roudsari A.T., Basiri Z., Yasrebifar F., Shahdoust M., Eshraghi A., Mehrpooya M., Ataei S. (2018). Evaluating the Effect of Oral N-acetylcysteine as an Adjuvant Treatment on Clinical Outcomes of Patients with Rheumatoid Arthritis: A Randomized, Double Blind Clinical Trial. Rev. Recent Clin. Trials.

[25] Kang E.H., Ha Y.-J., Lee Y.J. (2020). Autoantibody Biomarkers in Rheumatic Diseases. Int. J. Mol. Sci..

[26] Abd-Elazeem M.I., Mohamed R.A. (2018). Neutrophil-lymphocyte and platelet-lymphocyte ratios in rheumatoid arthritis patients: Relation to disease activity. Egypt. Rheumatol..

[27] Boulos D., Proudman S.M., Metcalf R.G., McWilliams L., Hall C., Wicks I.P. (2019). The neutrophil-lymphocyte ratio in early rheumatoid arthritis and its ability to predict subsequent failure of triple therapy. Semin. Arthritis Rheum..

[28] Aletaha D., Neogi T., Silman A.J., Funovits J., Felson D.T., Bingham C.O., Birnbaum N.S., Burmester G.R., Bykerk V., Cohen M.D. (2010). 2010 Rheumatoid arthritis classification criteria: An American College of Rheumatology/European League Against Rheumatism collaborative initiative. Arthritis Rheum..

[29] Langille E. (2019). Optimized Oxidative Stress Protocols for Low-microliter Volumes of Mammalian Plasma. Bio-Protocol.

[30] Jansen E.H.J.M., Beekhof P.K., Cremers J.W.J.M., Viezeliene D., Muzakova V., Skalicky J. (2013). Short-Term Stability of Biomarkers of Oxidative Stress and Antioxidant Status in Human Serum. ISRN Biomarkers.

[31] Padureanu R., Albu C.V., Mititelu R.R., Bacanoiu M.V., Docea A.O., Calina D., Pădureanu V., Olaru G., Sandu R.E., Malin R.D. (2019). Oxidative Stress and Inflammation Interdependence in Multiple Sclerosis. J. Clin. Med..

[32] Patsoukis N., Papapostolou I., Zervoudakis G., Georgiou C., Matsokis N.A., Panagopoulos N.T. (2005). Thiol redox state and oxidative stress in midbrain and striatum of weaver mutant mice, a genetic model of nigrostriatal dopamine deficiency. Neurosci. Lett..

[33] Colombo G., Clerici M., Garavaglia M.E., Giustarini D., Rossi R., Milzani A.D.G., Dalle-Donne I. (2016). A step-by-step protocol for assaying protein carbonylation in biological samples. J. Chromatogr. B.

[34] Spanidis Y., Goutzourelas N., Stagos D., Mpesios A., Priftis A., Bar-Or D., Spandidos D., Tsatsakis A.M., Leon G., Kouretas D. (2015). Variations in oxidative stress markers in elite basketball players at the beginning and end of a season. Exp. Ther. Med..

[35] Liu J., Yeo H.C., Doniger S.J., Ames B.N. (1997). Assay of Aldehydes from Lipid Peroxidation: Gas Chromatography–Mass Spectrometry Compared to Thiobarbituric Acid. Anal. Biochem..

[36] Keles M., Taysi S., Sen N., Aksoy Y., Akçay F. (2001). Effect of corticosteroid therapy on serum and CSF malondialdehyde and antioxidant proteins in multiple sclerosis. Can. J. Neurol. Sci. J. Can. Sci. Neurol..

[37] Janaszewska A., Bartosz G. (2002). Assay of total antioxidant capacity: Comparison of four methods as applied to human blood plasma. Scand. J. Clin. Lab. Investig..

[38] Demirci S., Demirci S., Kutluhan S., Koyuncuoglu H.R., Yurekli V.A. (2015). The Clinical Significance of the Neutrophil-to-Lymphocyte Ratio in Multiple Sclerosis. Int. J. Neurosci..

[39] Koffeman E.C., Genovese M., Amox D., Keogh E., Santana E., Matteson E.L., Kavanaugh A., Molitor J.A., Schiff M.H., Posever J.O. (2009). Epitope-specific immunotherapy of rheumatoid arthritis: Clinical responsiveness occurs with immune deviation and relies on the expression of a cluster of molecules associated with T cell tolerance in a double-blind, placebo-controlled, pilot phase II trial. Arthritis Rheum..

[40] Chen M.-H., Chou C.-T., Hou M.-C., Tsai C.-Y., Huang Y. (2020). Low but Long-lasting Risk of Reversal of Seroconversion in Patients with Rheumatoid Arthritis Receiving Immunosuppressive Therapy. Clin. Gastroenterol. Hepatol..

[41] Avci B.Ş., Avci A., Dönmez Y., Kaya A., Gülen M., Özer A.I., Bulut A., Koç M., Nazik H., Satar S. (2020). The Effectiveness of Neutrophil-Lymphocyte Ratio in Predicting in-Hospital Mortality in Non-ST-Elevation Myocardial Infarction. Emerg. Med. Int..

[42] Gao K., Zhu W., Liu W., Ma D., Li H., Yu W., Wang L., Cao Y., Jiang Y. (2019). Diagnostic value of the blood monocyte-lymphocyte ratio in knee osteoarthritis. J. Int. Med Res..

[43] Taşoğlu I., Çiçek Ö.F., Lafcı G., Kadirogulları E., Sert D.E., Demir A., Cavus U., Colak N., Songür M., Hodo B. (2014). Usefulness of Neutrophil/Lymphocyte Ratio as a Predictor of Amputation after Embolectomy for Acute Limb Ischemia. Ann. Vasc. Surg..

[44] Taşoğlu Ö., Bölük H., Onat Ş.Ş., Özgirgin N., Taşoğlu I. (2016). Is blood neutrophil-lymphocyte ratio an independent predictor of knee osteoarthritis severity?. Clin. Rheumatol..

[45] Panni R.Z., Lopez-Aguiar A.G., Liu J., Poultsides G.A., Rocha F.G., Hawkins W.G., Strasberg S.M., Trikalinos N.A., Maithel S., Fields R.C. (2019). Association of preoperative monocyte-to-lymphocyte and neutrophil-to-lymphocyte ratio with recurrence-free and overall survival after resection of pancreatic neuroendocrine tumors (US-NETSG). J. Surg. Oncol..

[46] Li K.J., Xia X.F., Su M., Zhang H., Chen W.H., Zou C.L. (2019). Predictive value of lymphocyte-to-monocyte ratio (LMR) and neutrophil-to-lymphocyte ratio (NLR) in patients with esophageal cancer undergoing concurrent chemoradiotherapy. BMC Cancer.

[47] Xu N., Tang X.-F., Yao Y., Zhao X.-, Chen J.-, Gao Z.-, Yang Y.-, Gao R.-L., Xu B., Yuan J.-Q. (2018). Predictive value of neutrophil to lymphocyte ratio in long-term outcomes of left main and/or three-vessel disease in patients with acute myocardial infarction. Catheter. Cardiovasc. Interv..

[48] Chandrashekara S., Ahmad M.M., Renuka P., Anupama K.R., Renuka K. (2017). Characterization of neutrophil-to-lymphocyte ratio as a measure of inflammation in rheumatoid arthritis. Int. J. Rheum. Dis..

[49] Uslu A.U., Kucuk A., Sahin A., Ugan Y., Yilmaz R., Gungor T., Bağçacı S., Kucuksen S. (2015). Two new inflammatory markers associated with Disease Activity Score-28 in patients with rheumatoid arthritis: Neutrophil-lymphocyte ratio and platelet-lymphocyte ratio. Int. J. Rheum. Dis..

[50] Fu H., Qin B., Hu Z., Ma N., Yang M., Wei T., Tang Q., Huang Y., Huang F., Liang Y. (2015). Neutrophil- and platelet-to-lymphocyte ratios are correlated with disease activity in rheumatoid arthritis. Clin. Lab..

[51] Sargın G., Senturk T., Yavasoglu I., Kose R. (2018). Relationship between neutrophil-lymphocyte, platelet-lymphocyte ratio and disease activity in rheumatoid arthritis treated with rituximab. Int. J. Rheum. Dis..

[52] Chen Q., Chen D.-Y., Xu X.-Z., Liu Y.-Y., Yin T.-T., Li N. (2019). Platelet/Lymphocyte, Lymphocyte/Monocyte, and Neutrophil/Lymphocyte Ratios as Biomarkers in Patients with Rheumatoid Arthritis and Rheumatoid Arthritis-Associated Interstitial Lung Disease. Med Sci. Monit..

[53] Peng Y.-F., Cao L., Zeng Y.-H., Zhang Z.-X., Chen D., Zhang Q., Zhu Y.-S. (2015). Platelet to lymphocyte ratio and neutrophil to lymphocyte ratio in patients with rheumatoid arthritis. Open Med..

[54] Tschopp J., Schroder K. (2010). NLRP3 inflammasome activation: The convergence of multiple signalling pathways on ROS production?. Nat. Rev. Immunol..

